# Apically extruded debris and irrigants during root canal filling material removal using Reciproc Blue, WaveOne Gold, R-Endo and ProTaper Next systems

**DOI:** 10.15171/joddd.2018.042

**Published:** 2018-12-19

**Authors:** Cangul Keskin, Evren Sarıyılmaz

**Affiliations:** ^1^Department of Endodontics, Faculty of Dentistry, Ondokuz Mayis University, Samsun, Turkey; ^2^Department of Endodontics, Faculty of Dentistry, Ordu University Ordu, Turkey

**Keywords:** Agar gel model, apical extrusion, retreatment, nickel-titanium

## Abstract

***Background.*** The present study aimed to compare the amount of apically extruded debris and irrigants produced by various nickel-titanium instruments.

***Methods.*** A total of 100 single-rooted mandibular premolar teeth were root canal treated and prepared for agar gel model. The root canal fillings were removed using Reciproc Blue, ProTaper Next, R-Endo, WaveOne Gold systems or hand instrumentation. The mean weights of apically extruded materials were calculated. Data were statistically analyzed using one-way ANOVA and post hoc Bonferroni tests.

***Results.*** Hand instrumentation resulted in significantly more debris and irrigants than other systems (P<0.05). The mean amount of apically extruded debris and irrigants produced by Reciproc Blue system was significantly greater than motordriven instruments (P<0.05). No significant difference was detected between ProTaper Next and WaveOne Gold systems (P>0.05), while they both produced significantly less apically extruded material than R-Endo system (P<0.05).

***Conclusion.*** All the instruments caused apical extrusion. ProTaper Next and WaveOne Gold systems were associated with significantly less apical extrusion.

## Introduction

Nonsurgical endodontic retreatment is generally the first treatment choice when an initial root canal procedure fails to eliminate the microbial infection of the root canal system. Nonsurgical retreatment aims to remove previous root canal filling materials in order to access apical foramen for further cleaning.^1^ Therefore, complete removal of root canal filling materials is crucial for a successful outcome of nonsurgical retreatment.^2^ During root canal filling removal procedures, extrusion of filling materials, tissue remnants, microorganisms and their by-products, dentin chips, and irrigation solutions beyond the apical foramen have been reported.^3^ Apical extrusion of foreign materials from an apical foramen has been associated with periapical inflammation, flare-ups, postoperative pain, delay in periapical healing, and long-term failure.^4-6^ Several techniques have been developed to remove gutta-percha from root canal systems including ultrasonics, lasers, hand files, nickel-titanium (NiTi) systems and solvents.^7-10^

Many available root canal preparation instruments and techniques have been associated with various degrees of apical extrusion. Reciprocating systems have been associated with less apical extrusion during root canal filling removal compared to rotary retreatment systems and hand files.^11,12^ WaveOne Gold (Dentsply Sirona, Ballaigues, Switzerland) is a novel reciprocating single-file instrument that replaced the previous WaveOne instruments and combines the metallurgical improvements of gold-wire thermal treatment to increase the elasticity and reciprocating motion. The WaveOne Gold instrument has a parallelogram cross-sectional shape with two 85° cutting edges, which enhances both cutting ability and debris removal.^13^ Reciproc Blue (VDW, Munich, Germany) is another novel single-file reciprocating instrument manufactured using thermomechanical treatment, which improves its fatigue resistance compared to its predecessor.^14^ The manufacturer of Reciproc Blue claims that this R25 Blue instrument could be used for retreatment such as Reciproc R25. According to our literature research, no studies have determined the amount of apically extruded debris and irrigants produced when WaveOne Gold and Reciproc Blue instruments were used during retreatment. The aim of this study was to evaluate the weight of apically extruded debris and irrigants during root canal filling removal using Reciproc Blue R25, WaveOne Gold Primary, R-Endo, ProTaper Next systems and hand instrumentation using the agar gel model. The null hypothesis was that there would be no differences among the tested instruments regarding apically extruded debris and irrigants.

## Methods

The study protocol was approved by the Ethics Board of the Medical Faculty (382). A total of 100 extracted human mandibular premolar teeth, each with a single straight root, a single root canal, and a fully formed apex were selected. Prior to the experiments, all the teeth were examined visually and radiographically to exclude teeth with resorption, previous root canal treatment or calcification. The specimens were decoronated under water cooling, using an Isomet saw (Buehler, IL, USA), to standardize specimen lengths at 15 mm (15±0.72 mm). Working lengths (WL) were determined visually with a #10 K-file introduced into the root canal until its tip was visible at the major apical foramen, and subtracting 1 mm from this length. The root canals were chemomechanically prepared with hand files using a standardized preparation up to the #40 K-file. Sodium hypochlorite (NaOCl, 5.25%) was used for irrigation during and after instrumentation. Final irrigation was achieved with ethylenediaminetetraacetic acid (EDTA; 17%), NaOCl (5.25%) and distilled water. The root canals were dried with paper points and obturated using a warm vertical compaction technique (BeeFill 2in1, VDW, Munich, Germany). The root canal walls were coated with a thin layer of sealer (AH Plus, Dentsply Sirona, Konstanz, Germany), and a 40.02 gutta-percha master cone (Aceone-Endo, Aceonedent Co. Geongg-Do, Korea) was inserted into the root canal with tug-back to the WL. The sequential removal of thermoplasticized gutta-percha and vertical compaction were completed when the plugger (BeeFill Downpack, VDW) was 3–4 mm from the WL. The middle and coronal thirds of the root canals were obturated using a BeeFill Backfill unit. Radiographs were taken in both the buccolingual and mesiodistal directions to confirm root canal filling quality. The specimens were stored at 37°C with 100% humidity for 2 weeks to allow the complete setting of the sealer.

**Figure 1:**
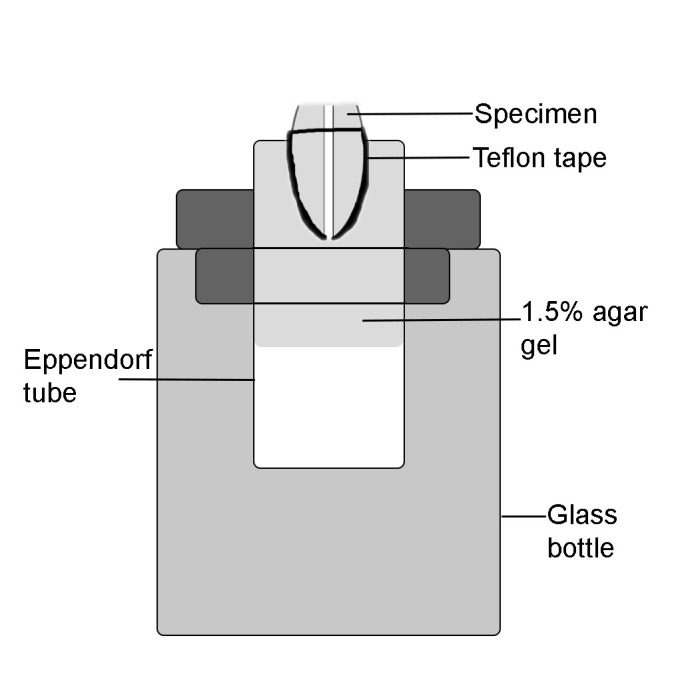
Schematic presentation of agar gel model. A root is positioned in Eppendorf tubes and immersed in 1.5% agar gel.

The specimens were randomly divided into five groups (n=20). All the specimen surfaces were wrapped with Teflon tape, leaving the apical foramen and coronal surface exposed. The specimens were numbered and weighed three times using an analytical balance with 10-^5^ g accuracy (Precisa XB 220A, Precisa Instruments, Dietikon, Switzerland). Agar gel (1.5%) was prepared as described in a previous study.^15^ Agar gel (1.5 mL) was injected into Eppendorf tubes, into which the specimens were positioned and fixed using cyanoacrylate. The tubes were inverted for specimens to be immersed into the agar (Figure 1). Following the gelation of the agar, each test apparatus was re-weighed three times. The weight of each apparatus was calculated by subtracting the first calculation from the second one. During root canal filling removal, the tubes were fixed in a glass bottle and a rubber sheet was placed around the coronal part of the roots for isolation. Groups were then identified and treated as outlined below.

**Hand instrumentation.** Coronal filling material was removed using Gates-Glidden drills. Hedström files (Dentsply Sirona) with 35, 30 and 25 sizes were used in a circumferential, quarter-turn push-pull, filing manner to remove filling materials until the WL was reached.

**Reciproc Blue.** A Reciproc Blue R25 instrument was used in the “Reciproc All” mode of a VDW silver endodontic motor (VDW, Munich, Germany) for root canal filling removal. The instrument was used in a slow in-and-out pecking motion with a 3-mm amplitude with a brushing motion against the root canal walls. Following three pecking movements, the instrument’s flutes were cleaned with sterile gauze impregnated with NaOCl (1%). This procedure was repeated until the instrument reached the WL.

**WaveOne Gold.** A WaveOne Gold Primary instrument was used in the “WaveOne All” mode of a VDW silver endodontic motor (VDW, Munich, Germany) for root canal filling removal. The instrument was used in a slow in-and-out pecking motion with 3-mm amplitude with a brushing motion against the root canal walls. Following three pecking movements, the instrument’s flutes were cleaned with sterile gauze impregnated with NaOCl (1%). This procedure was repeated until the instrument reached the WL.

**R-Endo.** R-Endo NiTi rotary retreatment files (Micro-Mega, Besancon, France), driven by the VDW silver endodontic motor set to 340 rpm, were used to perform the retreatment procedures. Re and R1 instruments were used at the coronal third, R2 was used in the middle third, and R3 was used in the apical third.

**ProTaper Next.** ProTaper Next instruments were driven by the VDW silver endodontic motor set at 300 rpm and 200 g/cm torque. X3 (30.07) instruments were used to remove the root canal filling from the coronal third and X2 (25.06) instruments were used in the full WL.

After reaching the WL, final apical preparation in all the groups was performed with #40 H-file. A total of 4 mL of 1% NaOCl was used as an irrigant during the root canal filling removal, and a final irrigation was performed using a 30-G Navi Tip needle (Ultradent, South Jordan, UT, USA) placed into the root canal 2 mm shorter than the WL. Following the final irrigation, the roots and surrounding Teflon tapes were removed from the tubes. Each instrument was used for one specimen and then discarded. One experienced operator (C.K.), who was an endodontist, performed all the root canal treatment and root canal filling retrieval procedures. The tubes were weighed and the amount of apically extruded debris and irrigants was calculated by subtracting the weight of the apparatus without teeth from the final weight. Weight analyses were performed by another experienced operator (E.S.) who was also blinded to the experimental groups.

All the measurements were made three times and the mean values were calculated. The normality of data distribution was confirmed with a Shapiro-Wilk test and data were analyzed with one-way ANOVA and post hoc Bonferroni tests at 95% level of significance using SPSS 21.0 (SPSS Inc., Chicago, IL, USA).

## Results

All the tested techniques produced measurable amounts of extruded debris and irrigants. The mean and standard deviation values for each experimental group are presented in Table 1. The hand instrumentation group produced the greatest amount of debris and irrigants compared to the other instruments (P<0.05). The Reciproc Blue system produced significantly more apically extruded debris and irrigants than R-Endo, WaveOne Gold and ProTaper Next systems (P<0.05). No significant difference was observed between the ProTaper Next and WaveOne Gold systems (P>0.05), while they produced significantly less apically extruded debris and irrigants than R-Endo system (P<0.05).

**Table 1:** Mean values and standard deviations of the amount of apically extruded debris and irrigation solutions in the experimental groups (×10^-2^ g)

	**Hand instrumentation**	**Reciproc Blue**	**WaveOne Gold**	**R-Endo**	**ProTaper Next**
**Mean weight of apically extruded debris**	0.72 ± 0.22^a^	0.54 ± 0.12^b^	0.30 ± 0.09^c^	0.41 ± 0.18^d^	0.32 ± 0.12^c^

Different superscript letters in the same line indicate significant difference (P<0.05).

## Discussion

During endodontic procedures, extrusion of debris, gutta-percha, sealer, and tissue remnants beyond the apical foramen has been associated with flare-ups, which is an unpleasant situation for both patients and clinicians.^5,6^ The apically extruded debris produced by the ProTaper Next and R-Endo instruments has been previously investigated in the literature;^16^ however, to our knowledge, there are no data on the amounts of apically extruded debris and irrigants produced by Reciproc Blue and WaveOne Gold systems during retreatment. The results of the present study showed that all the tested rotary and reciprocating instruments led to the apical extrusion of debris and irrigants to some degree in accordance with the previous literature.^10,11^ ProTaper Next and WaveOne Gold instruments caused significantly less apical extrusion than R-Endo and Reciproc Blue files. Therefore, the null hypothesis was refuted.

In the empty tube model, the apex is suspended in the air with no back pressure.^15^ However, periapical and/or granulation tissues might limit the amount of extruded materials in clinical situations.^17^ The present study used the agar gel method to simulate periapical tissues. Lu et al reported that a 1.5% agar gel model shows a similar density and provides resistance similar to periapical tissues to apically extruded materials.^15^ Therefore, the authors suggested that the use of this model is a more accurate method for the evaluation of extruded debris instead of the empty tube model.^15^ However, the agar gel model has some limitations since it does not represent all the periapical conditions, mainly because the thickness of the agar gel around the apex is standardized. The density of periapical tissues in cases requiring retreatment differ in that some cases present with periapical bone loss, whereas others present with granulation tissue, cysts, or periodontal ligament. Furthermore, in the agar gel model, it was not possible to distinguish root canal fillings from irrigation solutions. It has been shown that the extrusion of debris, gutta-percha, or irrigation solutions can lead to flare-ups.^6^ In the present study, following the root canal filling removal, extruded materials, which were mainly irrigation solution in volume and gutta-percha particles within the debris, were inspected.

Reciproc Blue instruments are manufactured identical to their predecessor apart from the use of thermomechanically treated alloys, and are operated within the same parameters as Reciproc. Thermomechanical treatment enhances an instrument’s mechanical properties, such as flexibility and cyclic fatigue resistance.^14^ Reciproc has been reported to produce a significantly greater amount of apically extruded debris when compared to the ProTaper Next in a recent study.^16^ In the present study, the mean amount of apically extruded debris and irrigant produced by ProTaper Next system was significantly lower than that of the Reciproc Blue system. The results of the present study supported the findings reported by Topçuoğlu et al,^16^ despite the metallurgical differences between Reciproc Blue and Reciproc files. Reciprocating movements push the debris towards the apex, whereas rotational movements favor the coronal transportation of debris.^18,19^ However, in the present study, two reciprocating instruments produced significantly different amounts of apically extruded debris and irrigants. One recent study evaluated the amount of apically extruded debris produced by WaveOne Gold system and reported that it produced significantly less apically extruded debris than the WaveOne system.^20^ Apart from the instrument movement, various factors have been shown to contribute to apical extrusion, such as the core mass, taper and cross-sectional shape of the instrument and final apical diameter of minor apical foramen.^11,19-21^ In the present study the root canals were further prepared with #40.02 H-file in circumferential motion in accordance with the prepared dimension of root canals prior to root canal fillings. Therefore, the final apical diameters of root canals were standardized. However, each instrument used in the present study had a different cross-sectional design; Reciproc Blue has an S-shape, WaveOne Gold has a parallelogram shape, ProTaper Next has a rectangular shape and R-Endo has a modified-triangular cross-sectional shape. WaveOne Gold files exhibit offset cross-sections similar to ProTaper Next files, which provides space for improved cutting, loading, and transportation of debris in the coronal direction.^22^ The offset cross-section and reduced taper might account for the reduced amount of debris and irrigant extrusion observed in the WaveOne Gold group compared to the Reciproc Blue group. Further studies are warranted to investigate the amount of residual root canal filling and retreatment time of the tested rotary and reciprocating systems to give clinicians information about the efficacy of these novel instruments for retreatment cases.

## Conclusion

Within the limitations of the present study, all the instruments produced apically extruded debris and irrigants. The ProTaper Next and WaveOne Gold systems were associated with significantly less apically extruded debris and irrigants than the R-Endo and Reciproc Blue instruments.

## Acknowledgments

The authors have no conflicts of interest to declare.

## Authors’ contributions

Cangül Keskin and Evren Sarıyılmaz are responsible for the design, experiment, data analysis, drafting and proofreading of the study.

## Funding

No funding information is available.

## Competing interests

Authors deny any conflict of interest.

## Ethics approval

The study protocol was approved by ethical committee of clinical studies of faculty of medicine.
